# Serum Uric Acid and Nigral Iron Deposition in Parkinson’s Disease: A Pilot Study

**DOI:** 10.1371/journal.pone.0112512

**Published:** 2014-11-11

**Authors:** Tae-Hyoung Kim, Jae-Hyeok Lee

**Affiliations:** Department of Neurology, Pusan National University Yangsan Hospital, Research Institute for Convergence of Biomedical Science and Technology, Yangsan, Korea; Northeastern University, United States of America

## Abstract

**Background:**

Uric acid (UA) is an endogenous antioxidant which is known to reduce oxidative stress and also chelate iron ion. Recent studies have provided evidence that UA may play a neuroprotective role in Parkinson’s disease (PD). However, it is unknown whether UA relates to nigral iron deposition, which is a characteristic pathophysiological alteration in PD. The aim of this study was to determine the potential relationship of these two markers in patients with PD.

**Methods:**

A total of 30 patients of PD and 25 age- and gender- matched healthy controls underwent 3-Tesla MRI and laboratory tests including serum UA levels. We assessed iron levels by measuring phase shift values using susceptibility-weighted image. Mean phase shift values of the substantia nigra (SN), red nucleus, head of the caudate nucleus, globus pallidus, putamen, thalamus, and frontal white matter were calculated and correlated with serum UA levels.

**Results:**

Serum UA levels were significantly decreased in the PD patients than in the controls. Phase shift values in bilateral SN were significantly increased in the PD patients than in the controls. There was no significant correlation between serum UA levels and nigral phase shift values.

**Conclusions:**

As previous studies, low serum UA level and increased nigral iron content in the PD was reconfirmed in this study. However, we failed to find the relationship between these two markers. Our data suggest that serum UA may not be important determinant of nigral iron deposition in PD.

## Introduction

Uric acid (UA), the end product of purine metabolism, is a natural antioxidant that may reduce oxidative stress [1]. In particular, higher concentrations of UA may protect against the development or progression of neurodegenerative diseases [1]. Indeed, recent studies have indicated that higher serum uric acid level is associated with lower incidence and better prognosis of Parkinson’s disease (PD) [2], [3], [4]. Furthermore, UA is reduced in the substantia nigra (SN) of PD patients [5]. In experimental models of PD, the administration of UA was found to suppress oxidative stress and prevent against nigral cell death [6], [7].

Excessive iron accumulation in the brain is a major contributor of oxidative stress by means of Fenton reaction which produces toxic hydroxyl radicals [8]. Increased iron levels in the SN had been reported consistently by postmortem and *in vivo* studies in PD [8], [9]. Iron-induced oxidative stress has been implicated in the degeneration of dopaminergic neurons [8], [9]. Also, toxic iron can promote α-synuclein misfolding and aggregation contributing to the pathogenesis of PD [8], [9]. Therefore, antioxidants with iron-chelating ability could be a viable neuroprotective approach for treatment of PD [10].

UA has been shown to have iron chelating property by forming stable complexes with Fe^3+^, and diminishing the oxidizing potential of Fe^3+^ [11]. Consequently, manipulation of UA concentrations could be an effective disease-modifying therapy in PD. At present, however, it is unknown whether UA and brain iron deposition are related in PD patients.

The aim of this study was to determine whether serum UA relates to brain iron content in patients with PD. We assessed iron levels in the various brain regions by calculating phase shift values from susceptibility weighted imaging, which is proven method to measure brain iron concentration [12]. Correlative analysis between between serum UA and brain iron levels may provide further understanding of the role of these two factors in the pathogenesis of PD.

## Materials and Methods

### 1. Subjects

A total 30 patients with PD and 25 age- and gender-matched healthy controls were included in this retrospective study. Data were collected from electronic medical records. All patients were diagnosed according to the UK Brain Bank criteria. Exclusion criteria for both PD patients and healthy controls were as follows: vegetarians, taking thiazide diuretics, suffering from renal disease, gout, acute medical illness, cancer, other neurological disorders, and subjects with microvascular lesions found on the brain MRI. Severity of disease and motor symptoms were assessed by the Hoehn and Yahr (H & Y) stage and the motor section of the Unified Parkinson’s Disease Rating Scale (UPDRS III) during the practically defined ‘OFF’ state. Serum UA levels were measured by an enzymatic colorimetric test from venous blood samples. The study was carried out in accordance with the Declaration of Helsinki and the protocol for this retrospective study was approved by Institutional Review Board, Pusan National University Yangsan Hospital with waiver of consent.

### 2. MRI acquisition and analysis

All patients and controls participating study underwent MRI using a 3-Tesla system (Siemens, Verio) equipped with a 12-channel head coil. The SWI data were acquired using flow-compensated 3D GRE sequences with an integrated Parallel Acquisition Techniques (iPAT) factor of 2. SWI images were taken parallel to the anterior–posterior commissural line with the following parameters: TR = 28 ms, TE = 20 ms, flip angle = 15°, matrix size = 320×260, field of view = 220×178 mm^2^, slice thickness = 2 mm. Both magnitude and phase images were acquired, but only phase data were used for analysis.

Regions of interest (ROIs) were placed on the filtered phase images and manually drawn according to the anatomical structures, bilateral substiantia nigra, red nucleus, globus pallidus, putamen, head of caudate nucleus, thalamus, and frontal white matter (Figure S1). Polygon ROIs adjusted to the shape and size of structure were used, but the outermost pixels were avoided to minimize partial volume effect. For frontal white matter, circular ROIs were drawn. Data on each structure were obtained from two consecutive slices, except for SN, where the most well recognized slice was used. The final shift value for each structure was calculated by averaging four measurements. Phase shift value in this study is for the left hand system [12], which is positively correlated with iron levels. ROIs drawing and calculation of phase shift values were performed using SPIN software (www.mrinnovations.com, Magnetic Resonance Innovations, Inc., Detroit, MI, USA). To verify reproducibility of the findings, phase shift values were re-measured by the same rater on the same images of 10 patients and 10 control subjects. Intraclass correlation coefficients (ICC) showed highly consistent results between two measurements (Table S1).

### 3. Statistical analysis

The demographic data between PD patients and control subjects were compared using Mann-Whitney’s U test or Fisher’s exact test. Analysis of covariance was used to compare phase shift values and serum UA levels between two groups with correction for age and gender. Correlation analysis using Pearson’s correlation coefficient was performed to investigate the relationship between phase shift values, serum UA levels, and clinical data. Statistical significance was determined as *p<0.05*. All statistical analyses were performed using SPSS (Chicago, Illinois) version 18.0.

## Results

Clinical data and laboratory findings of PD patients and control subjects are summarized in Table 1. There were no statistically significant differences in age, gender, BMI and serum creatinine (Cr) levels between PD and controls. The serum UA levels were significantly lower in the PD group than in the control group (4.7±1.4 mg/dl vs. 5.7±1.5 mg/dl, *p = 0.001*) after adjusting for age and gender. This difference was also statistically significant when comparing each gender separately (male group, 5.3±1.4 mg/dl vs. 6.7±1.3 mg/dl, *p = 0.018*; female group, 3.8±1.0 mg/dl vs. 4.8±1.2 mg/dl, *p = 0.011*). In patients with PD, there was no significant association between serum UA and clinical parameters including UPDRS III score and disease duration.

**Table 1:** Clinical characteristics and MRI data.

	Parkinson’s disease	Controls	*P* value
N (males/females)	30 (19/11)	25 (12/13)	0.286^a^
Age (years)	57.6±6.8	56.2±6.5	0.289^b^
Disease duration (months)	19.8±12.7		
UPDRS-III motor score	24.5±8.4		
Hoehn-Yahr stage (I/II)	9/21		
BMI (kg/m2)	24.0±3.0	24.8±2.6	0.237^b^
Serum creatinine (mg/dl)	0.88±0.19	0.87±0.19	0.872^b^
Serum uric acid (mg/dl)	4.7±1.4	5.7±1.5	0.001^c^
Phase value (radian)			
Frontal white matter	−0.002±0.002	−0.002±0.002	0.657^c^
Caudate nucleus	0.033±0.013	0.031±0.013	0.430^c^
Globus pallidus	0.036±0.014	0.035±0.015	0.725^c^
Putamen	0.018±0.012	0.014±0.008	0.260^c^
Thalamus	0.001±0.004	0.000±0.004	0.267^c^
Substantia nigra	0.050±0.023	0.035±0.020	0.001^c^
Red nucleus	0.061±0.023	0.052±0.026	0.226^c^

a *p*-value is calculated from Fisher’s exact test.

b *p*-value is calculated from Mann Whitney’s U-test.

c *p*-value is calculated from ANCOVA with adjustment for age and gender.

**Figure 1:**
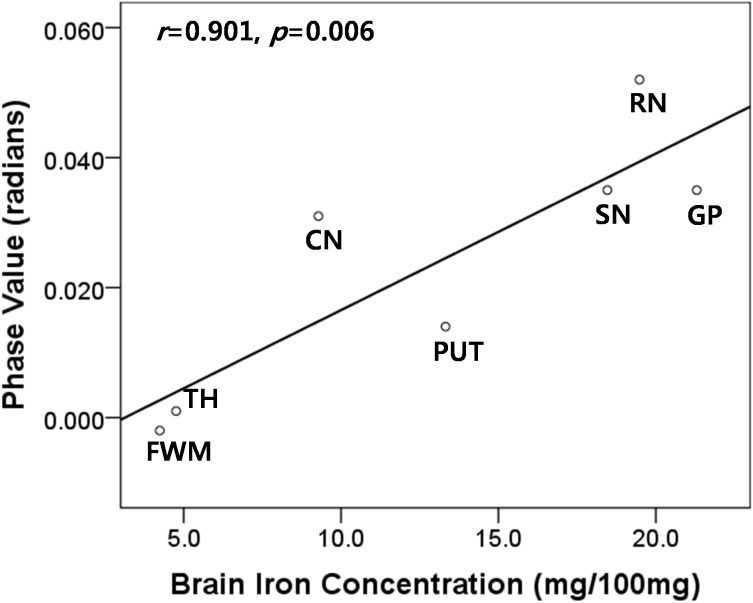
The correlation between the phase shift values in seven brain regions of healthy controls and postmortem iron concentrations, as reported by Hallgren and Sourander. CA = head of caudate nucleus; FWM = frontal white matter; GP = globus pallidus; PU = putamen; RN = red nucleus; SN = substantia nigra; TH = thalamus.

Spearman’s correlation analysis was applied to compare the phase shift values in the healthy controls and postmortem brain iron concentrations, as previously assessed by biochemical methods [13]. There was significant correlation between phase values and previously published iron concentrations (*r* = 0.901, *p = 0.006*), supporting the use of phase values as a viable method for estimating brain iron content (Figure 1).

Phase shift values of bilateral SN were significantly increased in the PD patients than in the controls (0.050±0.023 vs. 0.035±0.020, *p = 0.001*) after adjusting for age and gender. In the other brain structures, there was no significant difference of phase shift value between PD patients and control subjects. In the PD patient group, phase shift values in all regions of interest had no significant correlation with clinical parameters including UPDRS III score and disease duration.

**Figure 2:**
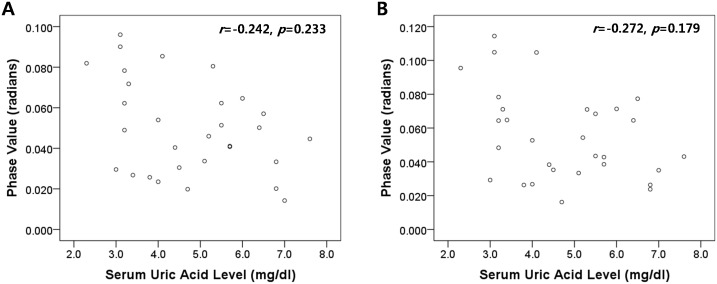
Scatter-plots of phase shift values of substantia nigra (A: bilateral SN, B: SN contralateral to the clinically more affected side) and serum uric acid levels in Parkinson’s disease subjects. The r and p values marked in the figure represent the results of Pearson’s partial correlation analysis adjusting for age, gender, UPDRS III score, and disease duration.

In patients with PD, there was no significant correlation between serum UA level and phase shift value of SN after adjustment for age, gender, UPDRS III score, and disease duration (*r* = −0.242, *p* = 0.233) (Figure 2). Furthermore, no significant correlation was found between serum UA levels and phase shift values of SN contralateral to the clinically more affected side (*r* = −0.272, *p* = 0.179). No significant correlation was found between two variables in control group after adjustment for age and gender (*r* = −0.159, *p = *0.468).

## Discussion

In this study, serum UA levels were significantly lower in the PD patients than control group. Also, phase shift values of SN which reflect iron content of brain tissue were significantly increased in patients with PD. These results are consistent with previous studies [3], [14], [15], suggesting both UA and iron may play an important role in the pathogenesis of PD. However, we failed to find the relationship between these two markers.

There is an abundance of clinical evidence that suggests that low UA levels are associated with the development and progression of a variety of neurological diseases, such as multiple sclerosis, Parkinson’s disease, and Alzheimer’s disease [1]. Also, associations between serum UA and brain MRI measures have been reported. In patients with multiple sclerosis, lower serum uric acid levels correlate with increased activity of disease and blood-brain barrier disruption on contrast-enhanced MRI [16], [17]. These observations raised the possibility that UA may play a protective role against neuroinflammation. To our knowledge, the present study is the first attempt to assess the potential relationship of serum UA and the MRI marker for PD.

So far, numerous studies had investigated possible relationship between brain iron deposition and serum biomarkers affecting iron homeostasis [15], [18]. Ceruloplasmin, a copper containing protein with ferroxidase activity, plays a significant role in iron homeostasis [19]. In a previous study, Jin L. et al. demonstrated that PD patients with reduced serum ceruloplasmin levels exhibited elevated nigral iron deposition as compared to those with normal serum ceruloplasmin levels [15]. Recent studies also have suggested higher serum cholesterol may be related to a lower occurrence and slower progression of PD [20]. One study found that higher serum cholesterol levels were associated with lower iron content in SN estimated from mean R2* values [18].

Due to its potent antioxidant properties, UA may have a neuroprotective effect by scavenging free radicals [1]. In addition, UA has metal chelating properties [1], [11]. The low UA concentration probably weakens the iron-chelating capacity and may expose the dopaminergic neurons to iron toxicity [6], [8]. In other words, UA may protect nigral cells through an UA-iron link. Based on these observations, we proposed UA as a possible biomarker of the iron dysregulation in PD. In the present study, however, we found no significant correlation between serum UA levels and phase shift values to quantify brain iron content. Our data suggest that serum UA may not be important determinant of nigral iron deposition in PD. Further studies with larger subjects are needed to elucidate the causal role of these two factors in the pathogenesis of PD.

There are several limitations in our study. First, sample size was relatively small to evaluate correlation between variables. Second, some of our results are different from several previous studies. Previous studies showed that iron content of SN in the PD patients was positively correlated with clinical disease severity such as UPDRS III motor score or disease duration [14], [15]. However, we did not found such correlation even when contralateral SN was analyzed. Third, cerebrospinal fluid (CSF) UA levels may better reflect the microenvironment of nigral neurons, although serum and CSF UA levels were highly correlated in current study [21], [22]. Moreover, serum UA levels are variable and were measured just once at the time of the study. Finally, we used manually-drawn ROIs in the selected slices, which may not reflect the actual total iron deposition in each structure. Other methodological issues such as sequence techniques, image resolution, and slice thickness may also contribute to inaccuracies in MRI-measured brain iron levels [23]. Iron measurement with phase value has some limitations. Phase values are markedly influenced by the difference in the magnetic susceptibility of the surrounding tissue besides the tissue iron content [24], [25]. Phase values can be compromised by its nonlocal and orientation dependent properties [26]. For these reasons, our data are insufficient to explain the link between iron and uric acid in the brain.

In conclusion, as previous studies, low serum UA level and increased nigral iron content in the PD was reconfirmed in this study. The lack of correlation between two factors suggests that serum UA levels may not be an indirect biomarker of the nigral iron alterations in PD. Further prospective studies with larger sample sizes should be conducted to confirm such a relationship.

## Supporting Information

Figure S1 **Illustration of phase images and the ROIs of substantia nigra, red nucleus, caudate nucleus, putamen, globus pallidus, thalamus, and frontal white matter.**
(TIF)

Table S1 **Intraclass correlation coefficients in patients with Parkinson’s disease and controls.**
(XLSX)
