# Supplementary figures and images for: Serum Uric Acid and Nigral Iron Deposition in Parkinson’s Disease: A Pilot Study
Source: PLoS One. 2014 Nov 11;9(11):e112512. doi: 10.1371/journal.pone.0112512 (PMC4227693; doi:10.1371/journal.pone.0112512)

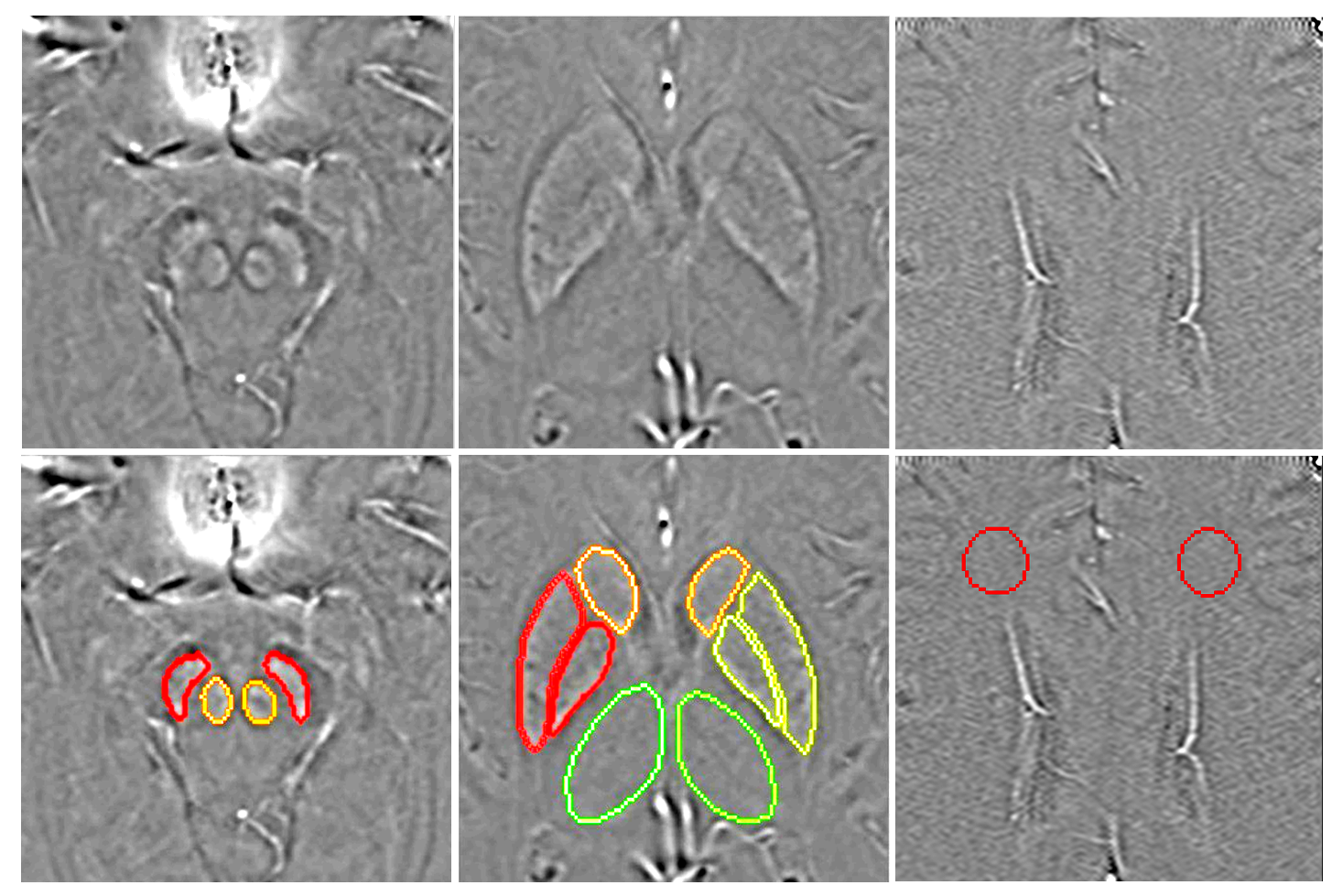

Supplement: Figure S1 — Illustration of phase images and the ROIs of substantia nigra, red nucleus, caudate nucleus, putamen, globus pallidus, thalamus, and frontal white matter. (TIF) [file pone.0112512.s001.tif]
